# *Acinetobacter baumannii* complex, national laboratory-based surveillance in South Africa, 2017 to 2019

**DOI:** 10.1371/journal.pone.0271355

**Published:** 2022-08-04

**Authors:** Olga Perovic, Adrian Duse, Vindana Chibabhai, Marianne Black, Mohamed Said, Elizabeth Prentice, Jeannette Wadula, Yesholata Mahabeer, K. Swe Swe Han, Ruth Mogokotleng, Wilhelmina Strasheim, Michelle Lowe, Sabelle Jallow, Husna Ismail, for GERMS-SA

**Affiliations:** 1 Centre for Healthcare-Associated Infections, Antimicrobial Resistance and Mycoses, National Institute for Communicable Diseases, Johannesburg, South Africa; 2 Division of Clinical Microbiology and Infectious Diseases, Faculty of Health Science, School of Pathology of the University of the Witwatersrand and the National Health Laboratory Service (NHLS), Johannesburg, South Africa; 3 National Health Laboratory Service, Charlotte Maxeke Johannesburg Academic Hospital, Microbiology laboratory, Johannesburg, South Africa; 4 Department of Medical Microbiology, University of Pretoria, Pretoria, South Africa; 5 National Health Laboratory Services, Tshwane Academic Division, Pretoria, South Africa; 6 Department of Medical Microbiology University of Cape Town and the National Health Laboratory Services, Groote Schuur Hospital, Cape Town, South Africa; 7 Chris Hani Baragwanath Academic Hospital, Soweto, South Africa; 8 Department of Medical Microbiology, National Health Laboratory Service, Inkosi Albert Luthuli Central Hospital, Durban, South Africa; 9 School of Laboratory Medicine and Medical Sciences, Nelson R. Mandela School of Medicine, University of KwaZulu-Natal, Durban, South Africa; University of Florida, UNITED STATES

## Abstract

**Objective:**

We aimed to provide an analysis of *A*. *baumannii* complex (ABC) isolated from blood cultures in South Africa.

**Materials and methods:**

ABC surveillance was conducted from 1 April 2017 to 30 September 2019 at 19 hospital sites from blood cultures of any age and sex. Organism identification was performed using the MALDI-TOF MS and antimicrobial susceptibility testing (AST), MicroScan Walkaway System. We confirmed colistin resistance with Sensititre, FRCOL panel, and selected for whole-genome sequencing.

**Results:**

During the study period, we identified 4822 cases of ABC, of which 2152 cases were from 19 enhanced surveillance sites were reported during the enhanced surveillance period (1 August 2018 to 30 September 2019). Males accounted for 54% (2611/4822). Of the cases with known age, 41% (1968/4822) were infants (< 1-year-old). Seventy-eight percent (1688/2152) of cases had a known hospital outcome, of which 36% (602/1688) died. HIV status was known for 69% (1168/1688) of cases, and 14% (238/1688) were positive. Eighty-two percent (1389/1688) received antimicrobial treatment in admission. Three percent (35/1389) of cases received single colistin. Four percent (75/2033) were resistant to colistin. At least 75% of the isolates (1530/2033) can be classified as extensively drug-resistant (XDR), with resistance to most antibiotics except for colistin. The majority, 83% (20/24), of the colistin-resistant isolates were of the sequence type (ST) 1. Resistance genes, both plasmid- and chromosomal- mediated were not observed. Although all isolates had, nine efflux pump genes related to antimicrobial resistance.

**Conclusion:**

Our surveillance data contributed to a better understanding of the natural course of *A*. *baumannii* disease, the patient characteristics among infants, and the level of resistance. At least two-thirds of the isolates were extensively drug-resistant, and four percent of isolates were resistant to colistin.

## Background

Antimicrobial resistance (AMR) threatens the efficacy of the successful treatment of infectious diseases and has public health implications at local, national, and global levels. AMR frequently occurs in microorganisms that are likely to be transmitted both, in the community and healthcare settings. *Acinetobacter* is an environmental organism that spreads efficiently in healthcare facilities and is a frequent cause of outbreaks.

According to the WHO priority pathogens list, the ESKAPE (*Enterococcus* spp., *Staphylococcus aureus*, *Klebsiella pneumoniae*, *Acinetobacter baumannii*, *Pseudomonas aeruginosa*, *and Escherichia coli or Enterobacter* spp.) are the most common healthcare-associated (HA) organisms with high rates of antibiotic resistance. In response, the National Institute for Communicable Diseases (NICD) established a laboratory-based antimicrobial resistance surveillance (LARS) program for ESKAPE organisms from sentinel tertiary hospital sites across South Africa.

While many species of *Acinetobacter* can cause human disease, *A*. *baumannii*, *A*. *pittii*, *A*. *nosocomialis*, and *A*. *calcoaceticus* account for about 80% of reported infections; making *Acinetobacter baumannii* complex (ABC) one of the most clinically important of HA pathogens [1].

The reservoir of ABC is soil and water, but it can be found on the skin of healthy people, especially hospitalized patients, and may survive in the healthcare environment for several days [2]. ABC may also colonize patients without causing infection or symptoms, such as in post-surgical wounds, catheter ports, tracheostomy sites, or open wounds. Clinical manifestations in patients with ABC range from mild to severe infections. Infections with ABC can cause bacteraemia, nosocomial pneumonia, meningitis, urinary tract infection, central venous catheter-related infection, and wound infection. ABC infection typically occurs in critically ill children and adult patients and can cause or contribute to the death [3].

The increasing prevalence of multidrug-resistant clinical strains of ABC is a major concern in healthcare settings as it causes public health problems due to limitations of therapeutic options, prolonged hospital stay, and its association with high morbidity and mortality [4].

ABC appears to be very effective at acquiring genetic material from other organisms, therefore this pathogen can rapidly develop antibiotic resistance resulting in multidrug-resistant (MDR), extensive drug-resistant (XDR), and pan drug-resistant ABC [5] resulting in severely limited available treatment options [6]. ABC can also be classified as carbapenem-resistant *A*. *baumannii* (CRAB); these isolates are usually multidrug-resistant thought CRAB, is listed as priority or critical number one on the WHO priority pathogens list for research and development of new antibiotics [7]. Optimal treatment should be made on a case-by-case basis by a clinician based on susceptibility testing results. XDR ABC strains remain commonly susceptible to polymyxins (colistin—polymyxin B) [8]. Combination therapy has been used with colistin, which includes tigecycline, carbapenems, or others. It has been debated whether colistin monotherapy or combination is superior. In a recent study, it was shown that colistin combination therapy with meropenem was associated with lower mortality, higher clinical and microbiological responses than colistin monotherapy, although a study done on pneumonia patients showed equivalent outcome [9, 10]. Nephrotoxicity risk was not increased with this combination treatment [10]. The emergence of colistin-resistant ABC is a major public health concern, as there are very limited novel antibiotics in the pipeline to treat XDR ABC infections [4, 11]. The mechanism of colistin resistance in ABC can be chromosomally encoded or plasmid-mediated. Among *A*. *baumannii* isolates, no plasmid-mediated *mcr1-5* genes have been detected from South Africa, however, the first detection of a plasmid *mcr*-4.3 gene encoding colistin resistance in *A*. *nosocomialis* from a clinical specimen was published recently [12, 13].

Using whole-genome sequencing of multidrug-resistant ABC provides a better understanding of resistant mechanisms and elucidates the genetic relationships among strains as well as source tracking. Colistin resistance was reported as chromosomally mediated with high MIC (≥16 μg/mL), however, expression of *mcr*1-10 leads to lower MIC (>2 μg/mL) or even susceptible values [14]. The colistin plasmid-mediated resistance is seldom detected in ABC as data from sequencing allows the characterization of a particular strain with the presence or absence of plasmid types.

### Study aim

We aimed to provide a clinical and microbiological characterization of *A*. *baumannii* complex bacteraemia from South Africa.

## Materials and methods

### Study design

This was a cross-sectional study. ABC surveillance was conducted from 1 April 2017 to 31 July 2018 under GERMS-SA, which is an active, laboratory-based surveillance program. We expanded the surveillance from 1 August 2018 to 30 September 2019 at 19 enhanced hospitals located in the Free State, Gauteng, KwaZulu-Natal, and the Western Cape provinces in South Africa using surveillance officers who collected demographic and clinical information (used GERMS-SA standardized case report form) from patients who met the case definition. In addition, we conducted data check audits from routine laboratories and added missing ones to the total number of ABC.

### Case definition

Isolation of ABC from blood cultures (BCs) of inpatients of any age and sex. Duplicate ABC isolates of the same organism obtained from the same patient within 21 days were regarded as duplicate isolates and excluded. All isolates were submitted to the Antimicrobial Resistance Reference Laboratory (AMRL) at the NICD.

### Laboratory testing

#### Phenotypic characterization

Isolates submitted from the testing laboratories were submitted to AMRL on Dorset slopes. We confirmed organism identification of ABC isolates with matrix-assisted laser desorption/ionization-time of flight mass spectrometry (MALDI-TOF MS) (Microflex, Bruker Daltonics, Germany). We performed antimicrobial susceptibility testing (AST) using the Microscan Walkaway System with the NM44 card (Beckman Coulter, USA). We confirmed colistin AST with the Sensititre® Vision® instrument (Trek Diagnostic Systems, UK) using the FRCOL panel (Separation Scientific, SA) as an alternate manual reading. The quality control strains E. coli ESCCO 33: NCTC 13846 (colistin positive) and *E*. *coli* ESCCO 01: ATCC27853 (colistin negative) were used in all AST assays. We interpreted the AST breakpoints using the Clinical Laboratory Standards Institute (CLSI) guidelines [15]. Tigecycline interpretation was excluded, as no breakpoint values were available by CLSI.

#### Genotypic characterization

Genomic DNA was extracted from pure bacterial cultures grown on 5% horse blood agar plates (Diagnostic Media Products, National Health Laboratory Service (NHLS), South Africa) using a crude boiling method, and used as a template for polymerase chain reaction (PCR) amplification [16, 17]. We used previously published methods and performed multiplex PCR to identify the *mcr*-1 to *mcr*-5 genes [18].

#### Whole-genome sequencing (WGS)

Genomic DNA was extracted for 24 isolates that had a colistin MIC ≥4 μg/mL and 14 colistin susceptible isolates (MIC ≤0.5 μg/mL) using QIAamp mini kit (Qiagen, Germany). We included 10mg/mL lysozyme (Sigma-Aldrich, USA) to ensure sufficient lysis. We measured the concentration of DNA using the Nanodrop 2000 spectrophotometer (Thermo Scientific, USA). Library preparation was done with the Nextera DNA Flex library prep kit (Illumina, USA) and sequencing was performed on the MiSeq platform (Illumina, USA) at a 2x300 bp read length at a 100x coverage. Raw sequencing reads were analyzed using the Jekesa pipeline (v1.0;). Briefly, Trim Galore! (v0.6.2; https://github.com/FelixKrueger/TrimGalore) was used to filter the paired-end reads (Q>30 and length >50 bp). *De novo* assembly was performed using SPAdes v3.13 and the assembled contigs were polished using Shovill (v1.1.0; https://github.com/tseemann/shovill) [19]. The multilocus sequence typing (MLST) profiles were determined using the MLST tool (v2.16.4; https://github.com/tseemann/mlst). Assembly metrics were calculated using QUAST (v5.0.2; http://quast.sourceforge.net/quast). The Center for Genomic Epidemiology web tools (https://cge.cbs.dtu.dk/services/) were used to construct the phylogenetic tree [Newick (NWK) file]. The exported NWK file was used in Phandango, Microreact (https://microreact.org/showcase) to visualize and edit the phylogenetic tree. The Resistance Gene Identifier (RGI) tool (v5.2.0) is hosted at the web portal of the Comprehensive Antibiotic Resistance Database (CARD) (https://card.mcmaster.ca/) and ResFinder (https://cge.cbs.dtu.dk/services/ResFinder/;) were used to describe the resistome of colistin-resistant *A*. *baumannii* from the assembled genome sequences [20, 21]. The sequences were submitted to Genbank with accession numbers JAKIIG000000000-JAKIJP000000000.

### Data management and analysis

Information on the GERMS-SA case report forms was captured on the GERMS-SA Microsoft Access Database (Microsoft Corporation, USA). We described surveillance data according to demographic and limited clinical variables. We performed statistical analysis using Stata version 15.1 (StataCorp LLC., USA). We used the Chi-square test to compare categorical variables and the Mann-Whitney Wilcoxon test to compare continuous variables. A *p*-value of <0.05 was considered statistically significant.

### Ethical considerations

This study was conducted in accordance with the Declaration of Helsinki and was approved by the Human Research Ethics Committee (HREC), Faculty of Health Sciences, University of the Witwatersrand, Johannesburg, South Africa (protocol number: M160667). Written consent was documented for all adult patients. For minor patients, parents or gradians gave written consent and was part of ethical approval by WITS HREC.

## Results

### Description of all ABC cases

**Fig 1:**
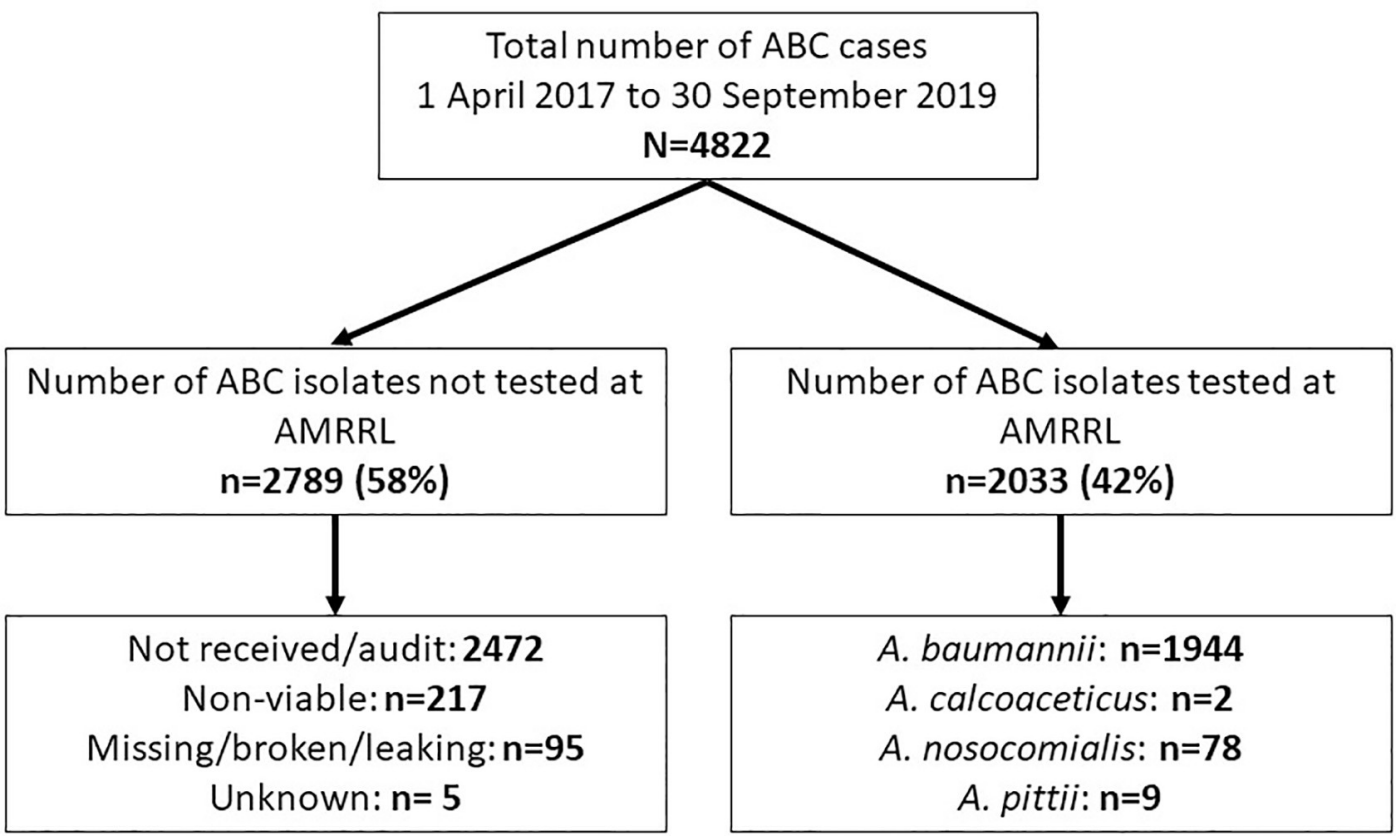
Cases of *Acinetobacter baumannii* complex (ABC) identified from South Africa from 1 April 2017 to 30 September 2019, N = 4822.

During the study period (1 April 2017 to 30 September 2019), we identified 4822 cases of ABC (Fig 1).

The majority of the cases were from Gauteng province (60%; 2917/4822) followed by KwaZulu-Natal (17%; 816/4822), the Free State (11%; 554/4822), and the Western Cape (11%; 523/4822) (Table 1). Males accounted for 54% (2611/4822) of cases and females accounted for 44% (2112/4822) (Table 1). About 48% (2336/4822) of cases were from the pediatric wards and 45% (2164/4822) from the adult wards (Table 1). Of the cases with known age, 41% (1968/4822) were infants (< 1-year-old) (Table 1).

**Table 1:** Characteristics of *Acinetobacter baumannii* complex (ABC) cases in South Africa from 1 April 2017 to 30 September 2019.

	Number of cases (N = 4822)	Percentage (%)
**Province**		
Gauteng	2 917	60
KwaZulu-Natal	816	17
Free State	554	11
Western Cape	523	11
Northern Cape	5	0.1
Eastern Cape	3	0.06
North West	2	0.04
Limpopo	1	0.02
Unknown	1	0.02
**Sex**		
Male	2 611	54
Female	2 112	44
Unknown	99	2
**Ward type**		
Adult (patients aged ≥18 years)	2164	45
Paediatric (patients aged <18 years)	2336	48
Unknown	322	7
**Age group**		
< 1 year	1 968	41
1–20 years	511	11
21–60 years	1651	34
>60 years	433	9
Unknown age	259	5

### Clinical characteristics of enhanced ABC cases

During the enhanced surveillance period (1 August 2018 to 30 September 2019), 2152 cases were identified. Bloodstream infections without focus accounted for 29% (628/2152) of cases. The median time from admission to bacteremia diagnosis was nine days (IQR 5–17 days). Seventy-eight percent (1688/2152) of cases had a known hospital outcome, of which 36% (602/1688) died (all-cause mortality) during hospital admission. Of the 602 cases that died, the all-cause 14-day mortality was 86% (518/602) and all-cause 30-day mortality was 94% (566/602). The median time from admission to the outcome was 15 days (IQR 7–30 days). HIV status was known for 69% (1168/1688) of cases, of which 14% (238/1688) were HIV-positive. Eighty-two percent (1389/1688) of cases received antimicrobial treatment during admission (Table 2). Of the cases that received antimicrobial treatment, 34% (475/1389) received colistin as a definitive treatment. Treatment regimens are described in Table 3.

**Table 2:** Clinical characteristics of *Acinetobacter baumannii* complex (ABC) cases with known patient outcomes in South Africa, 1 August 2018 to 30 September 2019.

	Total (N = 1688)*	Alive (N = 1086)	Died (N = 602)	
Characteristic	n (%)^a^	n (%)^b^	n (%)^b^	p-value
**Hospitalization**				
Median days in hospital (IQR)	15 (7–30)	24 (13–42)	12 (6–24)	<0.01
Median days in hospital before BSI (IQR)	9 (5–17)	10 (5–17)	8 (4–15)	<0.01
**Medical conditions**				
No	762	541 (71)	221 (29)	<0.01
Yes	726	451 (62)	275 (38)	
**Medical condition type**				
Diabetes mellitus	95	54 (57)	41 (43)	
Respiratory diseases	216	141 (65)	75 (35)	
Renal	120	69 (58)	51 (42)	
Cardio vascular	28	15 (54)	13 (46)	
Malignancy	72	36 (50)	36 (50)	
Other	343	219 (64)	124 (36)	
**Immune status**				
HIV-negative	930	634 (68)	296 (32)	0.08
HIV-positive	238	148 (62)	90 (38)	
**Referred from another healthcare facility**				
No	1171	779 (67)	392 (33)	0.21
Yes	460	291 (63)	169 (37)	
**Medical devices inserted during the current admission**				
No	57	51 (89)	6 (11)	<0.01
Yes	1513	995 (66)	518 (34)	
**Medical device type**				
Intravenous line	1051	712 (68)	339 (32)	
Central venous catheter	646	384 (59)	262 (41)	
Urinary catheter	527	304 (58)	223 (42)	
Drainage port	95	63 (66)	32 (34)	
Intra-arterial line	300	179 (60)	121 (40)	
Other	187	116 (62)	71 (38)	
**Antibiotics received during the current admission**				
No	181	107 (59)	74 (41)	0.02
Yes	1389	941 (68)	448 (32)	

*Numerators might not add up to the column total because of missing data

^a^ column percentage

^b^row percentage, interquartile range (IQR)

**Table 3:** Antimicrobial treatment of *Acinetobacter baumannii* complex (ABC) cases with known patient outcomes in South Africa, 1 August 2018 to 30 September 2019.

Antimicrobial treatment	Total (N = 1688)*	Alive (N = 1086)	Died (N = 602)
	n	n (%)^a^	n (%)^a^
Colistin monotherapy	35	28 (80)	7 (20)
Carbapenem monotherapy	219	126 (58)	93 (42)
Third- or fourth-generation cephalosporin monotherapy	20	17 (85)	3 (15)
Piperacillin-tazobactam monotherapy	65	33 (51)	32 (49)
Aminoglycoside monotherapy	25	17 (68)	8 (32)
Carbapenem plus third- or fourth-generation cephalosporin	21	17 (81)	4 (19)
Carbapenem plus aminoglycoside	40	21 (53)	19 (47)
Carbapenem plus aminoglycoside plus piperacillin-tazobactam	33	21 (64)	12 (36)
Carbapenem plus aminoglycoside plus other beta-lactam and beta-lactam inhibitor	46	18 (39)	28 (61)
Colistin plus carbapenem	167	125 (75)	42 (25)
Colistin plus carbapenem plus piperacillin-tazobactam	23	16 (70)	7 (30)
Colistin plus carbapenem plus aminoglycoside	38	33 (87)	5 (13)
Colistin plus carbapenem plus aminoglycoside plus other beta-lactam and beta-lactam inhibitor	44	36 (82)	8 (18)
Colistin plus carbapenem plus aminoglycoside plus piperacillin-tazobactam	27	20 (74)	7 (26)
Colistin plus other antimicrobial agents	43	33 (77)	10 (23)
Other antimicrobial agents	28	19 (68)	9 (32)

* not all treatment regimens were included

^a^row percentage, carbapenems (doripenem, ertapenem, imipenem, meropenem), 3rd or 4th generation cephalosporins (cefotaxime, ceftazidime, cefepime), aminoglycosides (amikacin, gentamycin, tobramycin), other beta-lactams + beta-lactam inhibitors (ampicillin-sulbactam, amoxicillin-clavulanate, cefotaxime-clavulanate, ceftazidime-clavulanate), other antibiotics (cefoxitin, fluoroquinolones (ciprofloxacin, levofloxacin), flagyl, piperacillin, trimethoprim-sulfamethoxazole)

### Characteristics of drug-resistant ABC

**Fig 2:**
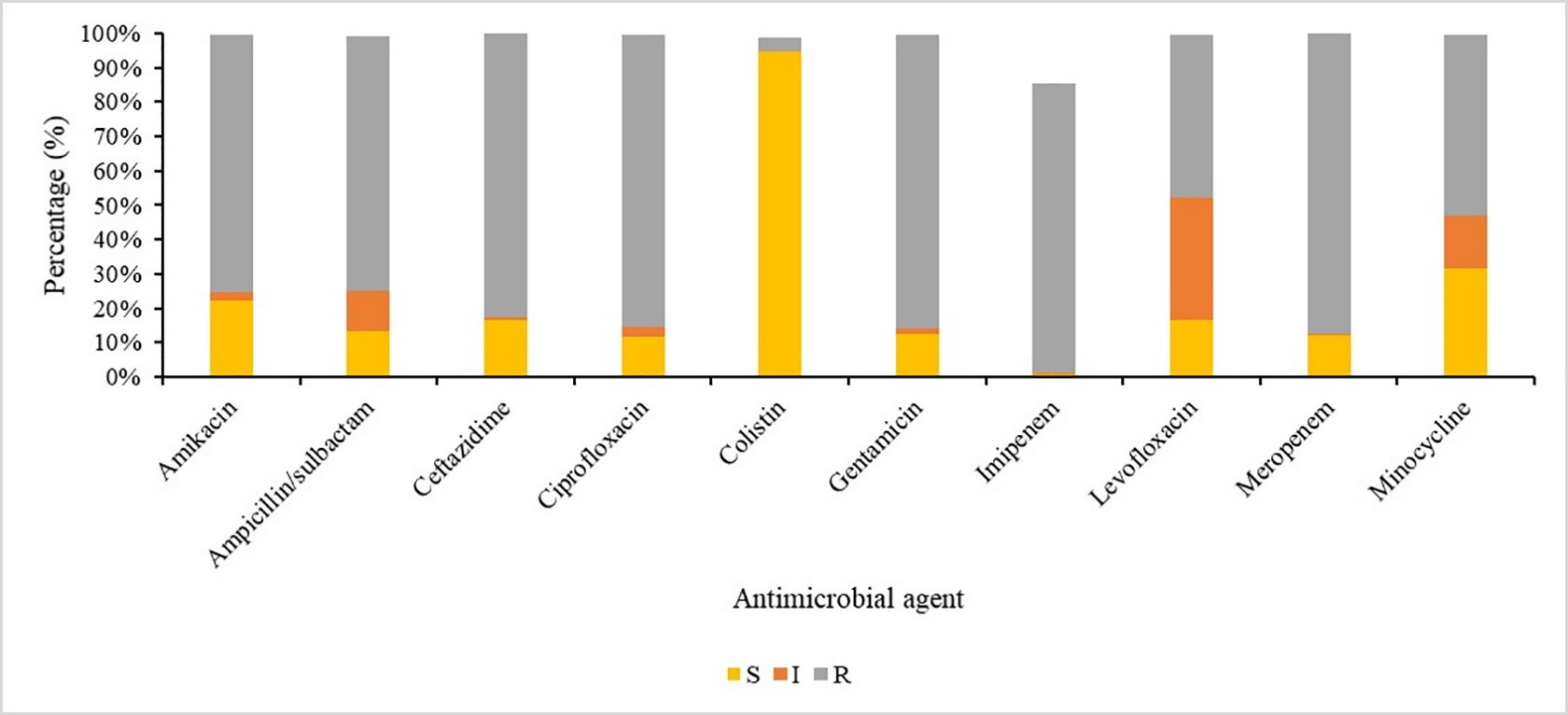
Antimicrobial susceptibility patterns of *Acinetobacter baumannii* complex (ABC) bloodstream isolates from South Africa from 1 April 2017 to 30 September 2019, n = 2033. Susceptible (S), Intermediate (I) and resistant (R).

**Fig 3:**
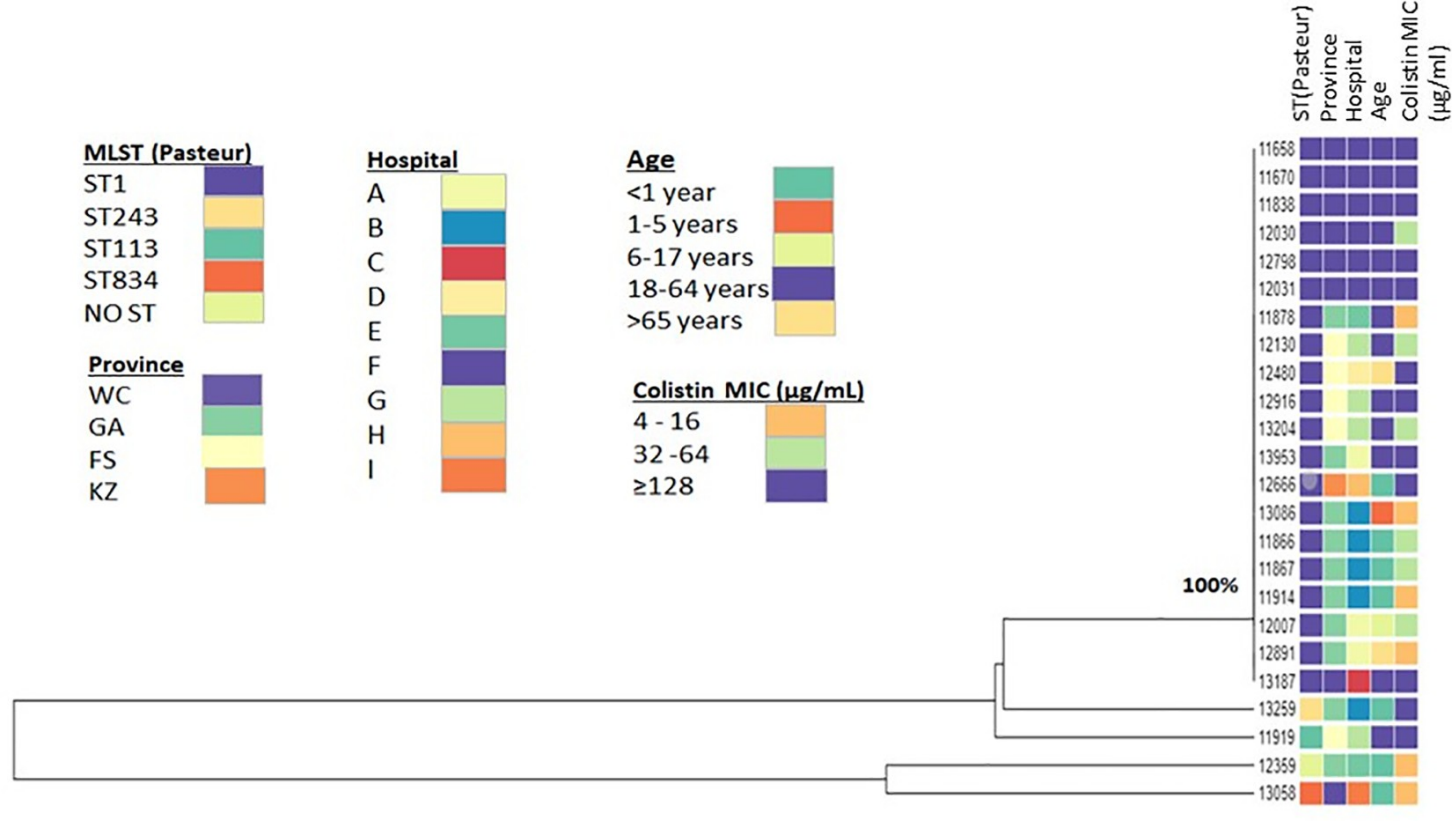
Phylogenetic comparison of colistin-resistant *Acinetobacter baumannii* bloodstream isolates from South Africa from 1 April 2017 to 30 September 2019, n = 24.

Of the 4822 cases of ABC, 42% (2033/4822) of isolates had a confirmed identification, of which 96% (1944/2033) were identified as *A*. *baumannii*, followed by 4% (78/2033) as *A*. *nosocomialis*. (Fig 1). *A*. *pittii* (9/2033) and *A*. *calcoaceticus* (2/2033) made up less than 1% of the total number of ABC isolates respectively (Fig 1). For the aminoglycosides, 75% (1530/2033) of isolates were resistant to amikacin and 85% (1738/2033) were resistant to gentamicin. For the third-generation cephalosporins, 82% (1676/2033) were resistant to ceftazidime; and for the carbapenems, 88% (1786/2033) of isolates were resistant to meropenem. Only four percent (75/2033) of isolates were resistant to colistin (Fig 2). At least 75% of the isolates (1530/2033) can be classified as extensively drug-resistant (XDR), being resistant to all listed relevant drug classes for *Acinetobacter* species, except for colistin [5, 22] (ref);. The majority, 83% (20/24), of the colistin-resistant isolates sequenced by WGS (n = 24), were of the sequence type (ST) 1 (Fig 3). Of the colistin-resistant isolates with WGS results, the majority (13/24 or 54.1%) were in adults (18–64 years old), followed by infants less than one year of age (7/24 or 29.1%) (Fig 3). The level of colistin resistance was distributed in the following ranges 4–16μg/ml in 6 (25%) isolates, 32–64 μg/ml in 10 (41.7%) isolates, and ≥128 μg/ml in 8 (33.3%) isolates (Fig 3). Antimicrobial minimum inhibitory concentration distribution was presented in Table 4.

**Table 4:** Antimicrobial minimum inhibitory concentration distribution of *Acinetobacter baumannii* complex (ABC) bloodstream isolates from South Africa from 1 April 2017 to 30 September 2019.

Antimicrobial agents (n =)				Number of isolates with MIC value of																MIC_50_	MIC_90_
	≤0.5	≤1	1	≤2	2	≥2	4	≥4	≤8	8	≥8	16	>16	32	>32	64	>64	128	>128		
Amikacin (2028)									440			10		48	1530					>32	>32
Gentamicin (2028)				212			47				1769									>8	>8
Tobramycin (2021)				449			25				1547									>8	>8
Ampicillin/Sulbactam (2017)									269			238	1510							>16	>16
Piperacillin (2024)									90			56				61	1817			>64	>64
Ceftazidime (2029)		23			132		129			55		14	1676							>16	>16
Cefepime (2029)		34			131		56			14		43	1751							>16	>16
Imipenem (1736)		17					3			87	1629									>8	>8
Meropenem (2019)		162			57		25				1775									>8	>8
Doripenem (227)		27			9			191												>4	>4
Ciprofloxacin (2027)	215		27		53	1732														>2	>2
Levofloxacin (2025)	310				24		725	966												4	>4
Tetracycline (2025)							307			138	1580									>8	>8
Minocycline (2022)							642			308	1072									>8	>8
Trimethoprim/Sulfamethoxazole (2009)				164				1845												>4/76	>4/76
Colistin (2001)	151		1319		458		8			11		9		6		12		14	13	1	2

### Molecular mechanism of resistance

To determine the molecular mechanism of resistance to colistin, we performed PCR on all 75 colistin-resistant isolates to investigate the presence of *mcr* genes. All 75 isolates were PCR-negative for the *mcr*-1 to *mcr*-5 genes. Whole-genome sequencing was performed on twenty-four colistin-resistant isolates from the laboratory-based surveillance with MIC≥ 4 μg/ml. For each drug class, at least 79% of isolates had known resistance genes (Table 3). The high proportion of observed phenotypic resistance was mainly due to the presence of resistance genes. Known resistance mechanisms in AMR databases were not observed for colistin and quinolones. For the rest of the antimicrobials, 83% (20/24) of isolates had at least one known resistance gene for all the drugs or drug classes listed in Table 3. The presence of known resistance genes in the remaining four isolates varied; one had resistance genes to aminoglycoside, β -lactams, and sulfonamide; another one had resistance genes to β -lactams and fluoroquinolone, and the last two had resistance genes for only β-lactams. A comparison of the 24 colistin-resistant with 14 colistin susceptible isolates, shows similar proportions of isolates with efflux pump genes (Table 5), indicating colistin resistance may not be mediated by these pumps.

**Table 5:** Summary of resistance genes present in the colistin-resistant *Acinetobacter baumannii* isolates per drug class (n = 24).

Drug class	AMR genes detected	Drugs	Percentage
**Aminoglycosides**	***aac(3)-Ia*, *aph(3’)-Ia*, *aph(3’’)-Ib*, *aph(6)-Id*, *arma***	gentamicin, sisomicin, kanamycin, streptomycin, *arma* (all aminoglycosides,except streptomycin)	**83%**
	*ant(3’’)-IIa*	streptomycin & spectinomycin	38%
	*ant(2’’)-Ia & aph(3’)*	Gentamicin, tobramycin, neomycin, paromycin	4%
**B-lactams**	***bla***_***ADC-79***_**, *bla***_***OXA-23***_ ***& bla***_***OXA-69***_	carbapenems, cephalosporins, penams	**83%**
	***bla***_***NDM-1***_	All b-lactams (carbapenems, cephalosporins, cephamycins, penams), except aztreonam	**79%**
	***bla***_*PER-7*_	Cephalosporins	
	***bla***_*ADC-4*_, ***bla***_*ADC-58*_, ***bla***_*ADC-68*_, ***bla***_*ADC-76*_, ***bla***_*OXA-51*_, ***bla***_*OXA-58*_ *&* ***bla***_*OXA-64*_	Carbapenems, cephalosporins, penams	4%
**Fluoroquinolones**	***AbaQ***	Fluoroquinolones	**92%**
**Fosfomycin**	***AbaF***	Fosfomycin	**88%**
**Macrolides**	***mphE***	Macrolides	**83%**
**Rifamycin**	***arr-2***	Rifamycin	**83%**
**Sulfonamide**	***sul1***	Sulfonamides	**83%**
	***sul2***		**88%**
**Tetracycline**	***tet(B)***	Tetracyclines	**79%**
**Diaminopyrimidines**	***dfrA1***	Trimethoprim	**83%**
**Bleomycin**	***BRP***_***MBL***_	Bleomycin only	**79%**
**Antibiotic target protection**	***msrE***	Macrolides, lincosamides, streptogramins, tetracyclines, oxazolidinone, phenicols, pleuromutilin,	**83%**
**Nucleoside**	***SAT-1***	streptothricin	**83%**
Phenicol	***cmlA5***	Chloramphenicol	**83%**
Acridine dye, disinfecting agents and intercalating dyes	*qacE (100%)*	Quaternary ammonium compounds (QAC)	54%
**Colistin** & Quinolones	**NO HITS**		
**Efflux pump**	***abeM*, *abeS*, *adeF*, *adeG*, *adeH*, *adeI*, *adeJ*, *adeK & AmvA***	Macrolides, lincosamides, streptogramins, tetracyclines, oxazolidinone, phenicols, pleuromutilin, fluoroquinolone, tetracyclines	**100%**
	***adeA*, *adeB*, *adeL*, *adeN*, *adeR & adeS***	Macrolides, fluoroquinolones, lincosamides, penems, carbapenems, cephalosporins, tetracyclin, rifamycin, Trimethoprim, Chloramphenicol, glycylcycline, tetracyclines	**92%**
	***adeC***	glycylcycline, tetracyclines	**83%**

## Discussion

In this report, we analyzed a high number of patients with ABC bacteremia from the national sentinel sites surveillance system and the majority of our cases were isolates of *Acinetobacter baumannii* acquired in hospital settings. Due to the high population density, most of the cases were identified in Gauteng province followed by KwaZulu-Natal and Western Cape. In our study, the highest distribution of ABC isolates was among children less than one-year-old, which is different, compared to a systematic review and meta-analysis by Lyu, where 11 studies were analyzed and population distribution was heterogeneous [23]. Our surveillance data showed a high distribution of ABC among children and is one of the most important findings, which is different from other studies.

As indicated, most of our cases were from children less than 1 year and the mortality rate was 36% out of the known number of patients. Similar results were observed in the study by Lyu, where the outcome of infants one month old was 30% with a low difference in the polymyxin compared to the non-polymyxin administration group. Gramatniece identified very few cases of *A*. *baumannii* bacteremia in neonates with a low mortality rate [3]. Later they notified an outbreak of ABC with a high colonization rate in the neonatal intensive care unit (NICU) [3]. HIV-positive status was not a significant risk factor for mortality (14%). Similar results were observed in another study on HIV-infected and uninfected adults from Thailand, where *Acinetobacter* spp was the most common cause of bacteraemia in HIV-uninfected patients [24].

A study by Balkhair showed that *A*. *baumannii* was the most frequent multidrug-resistant isolate with no resistance to colistin however; patients with *A*. *baumannii* isolates in blood had the worst-case fatality rate [25].

A group from Western Cape demonstrated an increase in numbers of *A*. *baumannii* isolates in a tertiary hospital from all specimen types and an increase in resistance to all antimicrobials [12]. They performed molecular typing and indicated that four strains were closely related within global clone ST1. All four had undescribed resistance to colistin, not defined previously.

We used a commercial broth microdilution method for colistin testing as recommended by ISO standard 20776–1, CLSI, and EUCAST guidelines. Matuschek evaluated the same method comparing Sensititre and two Micronaut products with ≥ 96% of essential agreement. Our results were based on the equivalent Sensititre method [22]. Using this method, we have identified colistin-resistant isolates, which we screened for chromosomal and plasmid *mcr*-mediated resistance and we have identified none. Although all the colistin-resistant isolates had at least nine efflux pump genes (Table 4), these genes were also equally present in colistin susceptible isolates, indicating little impact of these pumps on colistin resistance. Plasmid-mediated genes are not present and since efflux pumps do not seem to be the cause of the observed resistance, colistin resistance is most likely due to chromosomal mutations that are not present in the databases used for our analysis. However, we should monitor for detection of resistance genes in our isolates as they may present within a low level of resistance with MIC values at the breakpoint and even in susceptible. The role of efflux pumps in colistin resistance is suggested by several studies [12]. Mutations in *kpn*EF and *acr*AB, encoding components of efflux pumps, may lead to a two-fold decrease in the MIC of colistin and increase bacterial survival in the presence of a low concentration of polymyxins [26]. A Western Cape group performed WGS on four colistin-resistant and no *mcr*-1-5 genes were detected. Interestingly they found mutations on transporter family protein 1527N mutation (tripartite ATP-independent periplasmic transporter) not present in colistin-susceptible isolates [12]. In the study by Lin, efflux pump systems contributed to colistin resistance in *A*. *baumannii*. They found that deletion of *emr*AB gene increased colistin susceptibility, which indicates a role of efflux pumps in colistin susceptibility and is much less characterized compared to *Ade*ABC pumps [27]. The absence of plasmid-mediated and known chromosomal resistance mutations to colistin in the WGS data does not preclude novel or other chromosomal mutations not present in the current databases.

For the treatment of infections due to MDR ABC, combination treatment might be associated with bacterial eradication rather than monotherapy [4]. The same authors indicated that polymyxin B has a better pharmacokinetics and pharmacodynamics profile compared to colistin, however clinical evidence demonstrating pharmacology profiles is lacking; Balkhair et. al. surveyed the cause of 30-day mortality and demonstrated that in patients with ABC bacteremia who died, more than 50% were resistant to carbapenems and were adults [25]. Recently, polymyxin B demonstrated better pharmacokinetics and pharmacodynamics profiles and particularly featuring its active profile [4]. Shi indicated that based on the mortality of *Acinetobacter* pneumonia no evidence supported colistin plus carbapenem therapy over the colistin monotherapy [6].

Having said that colistin resistance is low in this surveillance report however Mendelson et al reported that prevalence in the animal sector is increasing and had reached 17% for *E*. *coli* in South Africa in the period from 1997–99, with a similar prevalence in 2014. However, *mcr*-1 was not detected, except in the period after 2015 [28]. Developing a One Health strategy for antibiotics stewardship would prevent the spread of resistance to colistin between sectors [29].

Sentinel sites included in the LARS were limited to tertiary level and academic hospitals; therefore, data generated and reported from the surveillance system does not necessarily represent infections from the whole population and antimicrobial susceptibility patterns from those in the more rural/smaller hospitals. Not all ABC isolates were sent to NICD for further testing. Key missing data such as clinical outcome data limit the generalizability of the findings and results need to be interpreted within the context of general surveillance limitations.

## Conclusion

Our surveillance data contributed to a better understanding of *A*. *baumannii* natural cause of disease, the patient’s characteristics particularly distribution among infants and the level of resistance to antibiotics among ABC isolates from the hospital sites. At least two-thirds of the isolates tested phenotypically were multidrug-resistant, with resistance to most antibiotics except for colistin; only 4% of isolates were resistant to colistin.

*Acinetobacter baumannii* is an important nosocomial pathogen posing a serious threat in South Africa, particularly in neonates and infants. *A*. *baumannii* can readily acquire numerous resistance mechanisms. This multi-drug and extended-drug resistant strains are extremely difficult to treat. Surveillance of this pathogen is important and should continue to track changes in virulence and antibiotic susceptibility profiles; this will ensure the availability of effective drug/s; recommendation of empiric therapy; and implementation of effective infection and prevention control measures.

## Supporting information

S1 Data (XLSX)
